# Caffeine Use: Association with Nicotine Use, Aggression, and Other Psychopathology in Psychiatric and Pediatric Outpatient Adolescents

**DOI:** 10.1100/tsw.2008.82

**Published:** 2008-05-22

**Authors:** Catherine A. Martin, Circe Cook, John H. Woodring, Gretchen Burkhardt, Greg Guenthner, Hatim A. Omar, Thomas H. Kelly

**Affiliations:** ^1^ Department of Psychiatry, University of Kentucky, Lexington, KY, USA; ^2^ Department of Pediatrics, University of Kentucky, Lexington, KY, USA; ^3^ Department of Behavioral Science, University of Kentucky, Lexington, KY, USA

**Keywords:** adolescence, risk behavior, caffeine, child health, human development, psychopathology

## Abstract

The objective of this study was to evaluate the relationship between caffeine use, other drug use, and psychopathology in adolescents, using self-report measures. The study group consisted of 132 adolescents (average age 14.01 ± 2.06 years, 52% female, 19% African American, 5% other categories, 76% Caucasian). Most (47%) were recruited from a child psychiatry clinic with emphasis on youth with disruptive disorders, with 35% from an adolescent pediatric clinic with emphasis on prevention of risk-taking behavior and 18% from a pediatric clinic for families with limited resources. Subjects were consecutively recruited before or after regular clinic visits. Consent was obtained from parents and assent from the youth. High caffeine consumption was associated with daily cigarette use; aggressive behavior; conduct, attention deficit/hyperactivity, and social problems; and increased somatic complaints in adolescents.

## INTRODUCTION

Up to 98% of adolescents regularly consume caffeine[1]. In normal adolescents, caffeine (e.g., up to 3 mg/kg) reduces reaction time and improves memory, attention, manual dexterity, and performance of vigilance tasks[2,3,4,5]. Higher doses of caffeine (>3 mg/kg) have been associated with difficulty sleeping, feeling tired in the morning, inattentiveness, nervousness, jitteriness, anxiety, nausea, and stomach ache[4,5,6,7,8].

Despite its ubiquitous use, few studies have examined the relationship between caffeine use and behavioral problems in adolescents. Caffeine has been reported to have a mildly positive effect on externalizing behaviors in Attention Deficit Hyperactivity Disorder (ADHD)[2], although there is little empirical evidence to support this claim[3,5]. In fact, available evidence suggests that caffeine may have a negative behavioral effect. When subjects are stratified by high or low caffeine consumption and are evaluated while consuming high doses of caffeine (>500 mg/day), low consumers report significantly greater adverse effects and have higher scores on the parent-completed Conners 10-item behavioral scale than high consumers[4,6]. Furthermore, the relationship between caffeine and other drug use in adolescents has received little attention. We performed a study to evaluate the relationship between caffeine use, other drug use, and psychopathology in adolescents.

## METHODS

The study group consisted of 132 adolescents (average age 14.01 ± 2.06 years, 52% female, 19% African American, 5% other categories, 76% Caucasian). Most (47%) were recruited from a child psychiatry clinic with emphasis on youth with disruptive disorders, with 35% from an adolescent pediatric clinic with emphasis on prevention of risk-taking behavior and 18% from a pediatric clinic for families with limited resources. Subjects were consecutively recruited before or after regular clinic visits. Consent was obtained from parents and assent from the youth.

### Variables

Caffeine use was assessed with self-report questionnaires in which the number and volume of caffeinated beverages consumed daily — including soft drinks, tea, and coffee — was obtained. Caffeine consumption was categorized as low (0–1 caffeinated beverages/day), medium (2–3 caffeinated beverages/day), or high (4 or more caffeinated beverages/day). Cigarette and alcohol use were assessed with self-report questionnaires that had been used previously in a longitudinal study of adolescent drug use, and assessed lifetime use and use in the last year, month, week, and day[7].

Adolescents completed Achenbach's Youth Self Report (YSR) and parents completed the Child Behavioral Checklist for ages 6–18 years (CBCL/6-18). The 2001 scoring techniques were used to generate the syndrome subscales, although older versions of the questionnaires (1991) were used. The Sensation Seeking Scale for Children (SSS-C) and the Children's Depression Inventory (CDI) were also administered.

### Statistical Methods

Two-sample t-tests were performed to determine if there were differences in caffeine use based on gender, cigarette and alcohol use, and clinic referral source. ANOVA with a Tukey HSD was used to determine if there were differences in scores on the subscales on the CBCL/6-18, YSR, SSS-C, and CDI based on high, medium, or low caffeine use. Due to the large number of bivariate comparisons, a conservative alpha level of 0.01 was used as the cutoff in determining statistical significance from the t-tests and ANOVA. All statistical analyses were conducted using SPSS for Windows, version 11 (SPSS, Inc. 2001).

## RESULTS

Ninety-one percent (120/132) of the subjects consumed caffeine daily; 9% (12/132) reported no caffeine use. Soft drinks were the main source of caffeine. Forty-two (32%) were low consumers of caffeine, 45 (34%) were moderate consumers, and 45 (34%) were high consumers — 13 (10%) consumed six or more caffeinated beverages daily.

High caffeine use was associated with daily cigarette use ($t\ [112] = 2.36$, $p \le 0.01$), but not with alcohol use. There were no differences in caffeine use by gender or clinic referral source.

Symptoms on the YSR varied significantly with caffeine intake, with the high caffeine intake group showing significantly higher scores than the moderate and/or low intake groups ($p \le 0.01$) on the aggressive behavior and conduct problem subscales (see Table 1) . Caffeine use was also associated with parent ratings on the CBCL/6-18, with the high caffeine intake group showing significantly higher scores than the moderate and/or low intake groups ($p \leq 0.01$) on aggressive behavior, attention, and social problem subscales and somatic complaint, attention deficit/hyperactivity, and conduct DSM (Diagnostic and Statistical Manual of Mental Disorders) subscales. Caffeine use was not associated with scores on the SSS-C or CDI.

**TABLE 1**
**ANOVA-Tukey HSD Comparisons of Achenbach Scores Between Low, Medium, and High Caffeine Users ($p \leq 0.01$)**

| Subscales | | Caffeine Consumption (Beverages/Day) | | | F | df | Group Difference |
|---|---|---|---|---|---|---|---|
| | | 0–1 | 2–3 | ≥4 | | | |
| Syndrome subscales | | | | | | | |
| Aggressive behavior | Youth | 6.86 ± 3.80 | 7.46 ± 5.00 | 11.40 ± 6.12 | 9.61 | 124 | High > medium and low |
| | Parent | 6.97 ± 7.30 | 9.67 ± 7.63 | 12.86 ± 7.97 | 6.17 | 127 | High > low |
| Attention problems | Parent | 5.09 ± 3.77 | 4.89 ± 3.57 | 7.36 ± 4.05 | 5.78 | 131 | High > medium and low |
| Social problems | Parent | 3.00 ± 3.19 | 4.51 ± 4.44 | 5.69 ± 4.81 | 4.48 | 132 | High > low |
| Somatic complaints | Parent | 3.00 ± 3.23 | 4.02 ± 4.32 | 5.09 ± 5.33 | 4.98 | 132 | High > medium and low |
| DSM-oriented subscales | | | | | | | |
| Somatic problems | Parent | 1.41 ± 1.79 | 2.75 ± 3.26 | 3.27 ± 3.09 | 4.89 | 129 | High > low |
| ADHD problems | Parent | 3.44 ± 2.96 | 3.56 ± 2.83 | 5.36 ± 3.06 | 5.89 | 132 | High > medium and low |
| Conduct problems | Youth | 4.33 ± 2.10 | 5.02 ± 3.14 | 7.12 ± 5.43 | 6.16 | 129 | High > medium and low |
| | Parent | 2.59 ± 3.89 | 4.59 ± 5.56 | 6.29 ± 6.60 | 4.74 | 126 | High > low |

## DISCUSSION

Contrary to prior studies suggesting that caffeine use by adolescents is benign or beneficial, we found that high caffeine consumption by adolescents was actually adversely associated with multiple externalizing symptoms based on both parent and child ratings, including tobacco cigarette use; somatic complaints; aggressive behavior; and conduct, social, and attention/ADHD problems (see Table 1). Based on these results, clinicians should consider the potential influence of caffeine intake on the externalizing problems of their patients. Given the ubiquitous use of caffeine, and that caffeine intake appears to be increasing and is associated with multiple adverse outcomes, a prospective longitudinal study to determine whether caffeine intake precedes or follows the emergence of other drug use and changes in externalizing symptoms is clearly warranted[5]. Furthermore, these results emphasize the need for further examination of the clinical effects of caffeine intake among ADHD adolescents.

## ACKNOWLEDGMENTS

This study was supported by NIDA grants number K08 DA00333 and DA05312, and University of Kentucky Medical Center Research Fund Grant 952.
